# Cold Plasma Modification of Cassava Starch–Gelatin Blend Films for Enhanced Mechanical Strength and Water Resistance in Sustainable Packaging

**DOI:** 10.3390/polym18202573

**Published:** 2026-10-19

**Authors:** Chonticha Srakam, Nanthicha Thajai, Pornchai Rachtanapun, Sarinthip Thanakkasaranee, Nuttapol Tanadchangsaeng, Patnarin Worajittiphon, Kittisak Jantanasakulwong

**Affiliations:** 1 Faculty of Agro-Industry, Chiang Mai University, Chiang Mai 50100, Thailand; chonticha.skm@gmail.com (C.S.); pornchai.r@cmu.ac.th (P.R.); sarinthip.t@cmu.ac.th (S.T.); 2 Nanoscience and Nanotechnology (International Program/Interdisciplinary), Faculty of Science, Chiang Mai University, Chiang Mai 50200, Thailand; nanthicha581@gmail.com; 3 Center of Excellence in Agro Bio-Circular-Green Industry (AgroBCG), Chiang Mai University, Chiang Mai 50100, Thailand; 4 College of Biomedical Engineering, Rangsit University, Pathumthani 12000, Thailand; nuttapol.t@rsu.ac.th; 5 Department of Chemistry, Faculty of Science, Chiang Mai University, Chiang Mai 50200, Thailand; patnarin.w@cmu.ac.th

**Keywords:** starch, gelatin, blended films, cold plasma, biopolymer, sustainable agriculture

## Abstract

A cold plasma-treated cassava starch–gelatin blended film (CS-GL) was developed to enhance the mechanical, thermal, and physical properties of biopolymer films for sustainable packaging applications. Cassava starch and gelatin were blended at an 80:20 ratio and treated using a cold plasma jet at 7 W with exposure times of 5–45 min and at plasma powers of 4–13 W for 15 min. Plasma treatment at moderate power and duration promoted structural interactions between starch and gelatin chains, as supported by FTIR by FT-IR and ^1^H-NMR, while excessive power and prolonged exposure caused depolymerization of the polymer chains. The tensile strength of the CS-GL films increased from 30 to 42 MPa at 7 W for 15 min, whereas the elongation at break decreased from 7.62% to 5.42%. The TGA results showed that the residual content increased with plasma power up to 10 W and with exposure time up to 45 min but decreased to 13 W owing to chain scission. DSC analysis showed an increase in the endothermic transition temperature under moderate plasma conditions and a broader transition with a larger amorphous fraction under excessive treatment. Plasma treatment increased the water contact angle while reducing swelling and solubility owing to the consumption of hydroxyl groups and network densification. SEM images showed surface smoothing and densification at moderate plasma power and surface roughening at high power owing to chain scission. The optimal conditions were identified as 7 W for 15 min, which provided the best balance between mechanical strength, thermal stability, and structural homogeneity. A moderately higher dose further improved water resistance. Cold plasma is an effective and controllable green modification technique for tailoring the mechanical and water-resistance properties of starch–gelatin biopolymer films, offering a promising basis for future development toward biodegradable packaging industry, pending further evaluation of migration safety, packaging regulation, biodegradation behavior, and storage stability.

## 1. Introduction

The growing demand for sustainable and environmentally friendly materials has driven considerable interest in biopolymer-based films as alternatives to conventional petroleum-derived plastics. Biopolymers are naturally derived polymers obtained from renewable biological sources including polysaccharides, proteins, and lipids. Unlike synthetic plastics, which are nonbiodegradable and contribute to persistent environmental pollution and greenhouse gas emissions, biopolymers are biodegradable, recyclable, nontoxic to humans and the environment, and cost-effective [1,2]. These characteristics make biopolymers highly attractive for applications in the food packaging, agriculture, and biomedical fields, where the need for green and sustainable materials is increasingly recognized. Among biopolymers, polysaccharides such as chitosan, cellulose, starch, alginate, and carrageenan have been extensively studied for the development of biodegradable and edible films and coatings [3,4]. Starch is one of the most widely used polysaccharides for film preparation because of its abundance, low cost, transparency, and film-forming capability. Cassava starch (CS), derived from cassava root (*Manihot esculenta*), is of special interest because of its high amylopectin content, which contributes to good film flexibility and clarity. However, starch-based films inherently exhibit poor mechanical properties and low water resistance, which are attributed to the presence of numerous hydroxyl groups that render the material hydrophilic [3]. These limitations restrict the broader application of starch films in packaging and other industrial uses and thus necessitate further modification or blending with complementary materials to improve film performance [4,5].

Proteins represent another important class of biopolymers with high potential for film fabrication. They exhibit favorable properties, including good mechanical strength and effective barrier properties against oxygen, odor, and oil under low-to-medium relative humidity conditions [3]. Gelatin (GL), an animal-derived protein obtained from the partial hydrolysis of collagen, is particularly attractive for blending with starch owing to its excellent film-forming ability, biocompatibility, and biodegradability. GL is composed of polypeptide chains with diverse functional groups, including amide, amino, and hydroxyl groups, that enable the formation of an extensive hydrogen-bonding network. These interactions between the polar groups of starch and gelatin promote intermolecular compatibility, resulting in a reinforced composite film matrix with enhanced cohesion and mechanical performance [4]. By leveraging the complementary properties of starch and GL, blended CS-GL films can achieve a more balanced combination of flexibility, strength, and barrier properties than films prepared from either component alone [6].

Despite the promising properties of CS-GL blended films, their practical application is still constrained by insufficient mechanical strength and hydrophilicity. Modification strategies have been explored to address these challenges and can be broadly categorized into chemical, enzymatic, and physical methods [5]. Among these, physical modification techniques are particularly attractive because they maintain the biological integrity and purity of the biopolymers without introducing chemical residues. Cold plasma treatment has emerged as a promising nonthermal physical modification technique, offering several advantages, including rapid processing, environmental friendliness, and the ability to induce targeted structural changes without direct contact with the material [7,8]. Cold plasma generates a mixture of reactive species, including ions, free radicals, ultraviolet photons, and electrons, which interact with the surface and bulk structures of biopolymers. These reactive species break existing chemical bonds, particularly O–H and C–OH bonds, generating reactive sites that can facilitate the formation of new covalent crosslinks between the polymer chains [9]. In the case of starch–gelatin blends, cold plasma promotes crosslinking between the hydroxyl groups of starch and the functional groups of gelatin, thereby creating a denser polymer network. Structural reorganization has been reported to significantly improve the mechanical, thermal, and barrier properties of biopolymer films [7,10,11]. However, the extent and nature of plasma-induced modifications are strongly dependent on the treatment parameters, particularly the plasma power and exposure time, which determine whether crosslinking or depolymerization mechanisms predominate. Although cold plasma has been separately applied to individual biopolymer systems such as starch or gelatin, its application to CS-GL blended films has not been sufficiently explored, particularly with respect to the optimization of plasma power and treatment duration.

Therefore, this study aims to investigate the effects of cold plasma treatment on the structural, mechanical, thermal, and physical properties of CS-GL blended films. Cold plasma was applied at plasma powers of 4, 5, 7, 10, and 13 W with a fixed treatment time of 15 min and at 7 W with varying treatment durations of 5, 10, 15, 30, and 45 min. The chemical structural changes were characterized using FT-IR and ^1^H-NMR spectroscopy, while the mechanical properties, thermal stability, water solubility, water swelling, water contact angle, and surface morphology were evaluated to assess the influence of plasma treatment conditions comprehensively. The findings of this study are expected to provide valuable insights into the design of cold plasma-modified biopolymer films with improved performance, offering a green and scalable approach for the development of next-generation biodegradable packaging materials.

## 2. Materials and Methods

### 2.1. Materials

CS (Dragon Fish brand) was purchased from the Ngchan Registered Ordinary Partnership in Bangkok, Thailand. Gelatin (GL, 250 bloom) was purchased from Lana Grow Chemical Co., Ltd. (Nonthaburi, Thailand).

### 2.2. Preparation of CS-GL Blend Films

CS (5% *w*/*w*) was dissolved in distilled water at 80 °C for 30 min, and GL (2% *w*/*w*) was separately dissolved in distilled water at 80 °C for 10 min. The two solutions were then blended at an 80:20 (CS/GL) ratio at 70 °C for 30 min to obtain the CS-GL film-forming solution. The blended solution was subsequently treated with cold plasma (Section 2.3), cast onto flat, leveled plates, and dried to form films of uniform thickness for further characterization.

### 2.3. Cold Plasma Treatment

Cold plasma treatment of the CS-GL solution was carried out using an argon plasma jet (50 Hz, 0.095 A, 230 V) with a fixed probe-to-sample distance of 15 cm, following the method of Ledari et al. [12]. Because plasma was applied to the CS-GL solution rather than to a solid, pre-formed film, reactive species could interact with polymer chains throughout the treated liquid volume rather than being confined to a surface layer, consistent with the plasma-activated liquid/plasma-activated water mechanism [7]. The plasma jet used an atmospheric-pressure cold plasma jet (APPJ/CAP jet) (nozzle inner diameter 1 mm) configuration with argon supplied at 1.2 L/min; the CS-GL solution 50 mL was placed in a 25 × 150 mm glass Petri dish and treated over a surface area of approximately 0.785 cm^2^. Two treatment series were investigated: the effect of plasma power at 4, 5, 7, 10, and 13 W for a fixed exposure time of 15 min, and the effect of exposure time at 5, 10, 15, 30, and 45 min at a fixed power of 7 W. The treatment conditions are summarized in Table 1.

### 2.4. Fourier-Transform Infrared Spectroscopy (FT-IR)

The chemical structures of the films were analyzed by FT-IR spectroscopy (FT/IR-4700; Jasco Corp., Tokyo, Japan) in the attenuated total reflectance (ATR) mode. The spectra were recorded over the range of 500 to 4000 cm^−1^ at a resolution of 16 cm^−1^. FT-IR spectra were recorded at a resolution of 16 cm^−1^. The spectra were used to evaluate changes in the major functional-group absorption bands associated with the polymer components.

### 2.5. Nuclear Magnetic Resonance Spectroscopy (NMR)

The molecular structures of the films were analyzed by ^1^H-NMR spectroscopy. Each sample was dissolved in dimethyl sulfoxide (DMSO) at a concentration of 0.01 g mL^−1^ and transferred to an NMR tube. The spectra were recorded using TopSpin 4.0.8 (Bruker BioSpin GmbH, Karlsruhe, Germany).

### 2.6. Tensile Properties

The tensile strength and elongation at break of the films were determined according to JIS K 6251-7 [13] using a universal testing machine (MCT-1150; A&D Company Limited, Tokyo, Japan). Film thickness was determined from the specimen dimensions recorded during tensile testing (used to calculate stress in MPa); the mean thickness (±SD) across all conditions was 0.161 ± 0.04, with no significant difference among treatments. The film specimens were 5 mm wide and 20 mm long and conditioned at 25 °C and 52% relative humidity (RH) for 48 h before testing. The measurements were repeated five times for each sample.

### 2.7. Morphology

The morphologies of the films were examined by scanning electron microscopy (SEM) using a JSM-IT200 instrument (JEOL Co., Ltd., Tokyo, Japan) at 15 kV. The samples were fractured in liquid nitrogen and dried at 50 °C for 24 h to remove moisture. The fractured samples were mounted on stubs and coated with gold prior to observation.

### 2.8. Differential Scanning Calorimetry (DSC)

DSC analysis was performed using a DSC823e instrument (Mettler Toledo, Columbus, OH, USA). About 5 to 10 mg of each sample was sealed in a 40 µL aluminum pan and heated from 0 to 200 °C at a rate of 10 °C min^−1^.

### 2.9. Thermogravimetric Analysis (TGA)

TGA was performed using a TGA/DSC3+ HT (Mettler Toledo, OH, USA). About 7 mg of each sample was placed in an alumina crucible and heated from 25 to 600 °C at a rate of 10 °C min^−1^.

### 2.10. Water Solubility

The water solubility of the films was evaluated based on dry weight loss after immersion. This method was adapted from Tongdeesoontorn et al. [6]. The film samples (2 × 2 cm) were dried at 60 °C for 24 h and weighed to obtain the initial weight (W_i_). The samples were immersed in 20 mL of distilled water at 25 °C and shaken for 24 h. After the water was removed, the final weights of the samples (W_f_) were recorded. The tests were performed in triplicate for each sample. Water solubility was calculated using Equation (1).

Water solubility (%) = [(W_i_ − W_f_)/W_i_] × 100 (1)

### 2.11. Water Swelling

Water swelling of the films was evaluated based on the weight gained after immersion. This method was adapted from ASTM D-570 [14]. The dried film (60 °C for 24 h) samples (2 × 2 cm) were weighed to obtain the initial weight (W_i_) and immersed in 20 mL of distilled water at 25 °C for 2 h. After removing surface water, the final weights of the samples (W_f_) were recorded. The tests were performed in triplicate for each sample. Water swelling was calculated using Equation (2).

Water swelling (%) = [(W_f_ − W_i_)/W_i_] × 100 (2)

### 2.12. Water Contact Angle

The water resistance of the films was evaluated based on their water contact angles. The film samples (1 cm × 1 cm) were mounted on glass. Water droplets were placed on the film surface and imaged every 1 min for 10 min using a drop-shaped analyzer (DSA30; Krüss, Hamburg, Germany). The tests were performed in triplicate for each sample.

### 2.13. Statistical Analysis

Data were analyzed using SPSS version 22. One-way analysis of variance (ANOVA) followed by Duncan’s multiple range test was performed to compare the means at a significance level of *p* < 0.05. The results are reported as the mean ± standard deviation (mean ± SD).

## 3. Results and Discussion

### 3.1. Fourier-Transform Infrared Spectroscopy (FT-IR)

**Figure 1:**
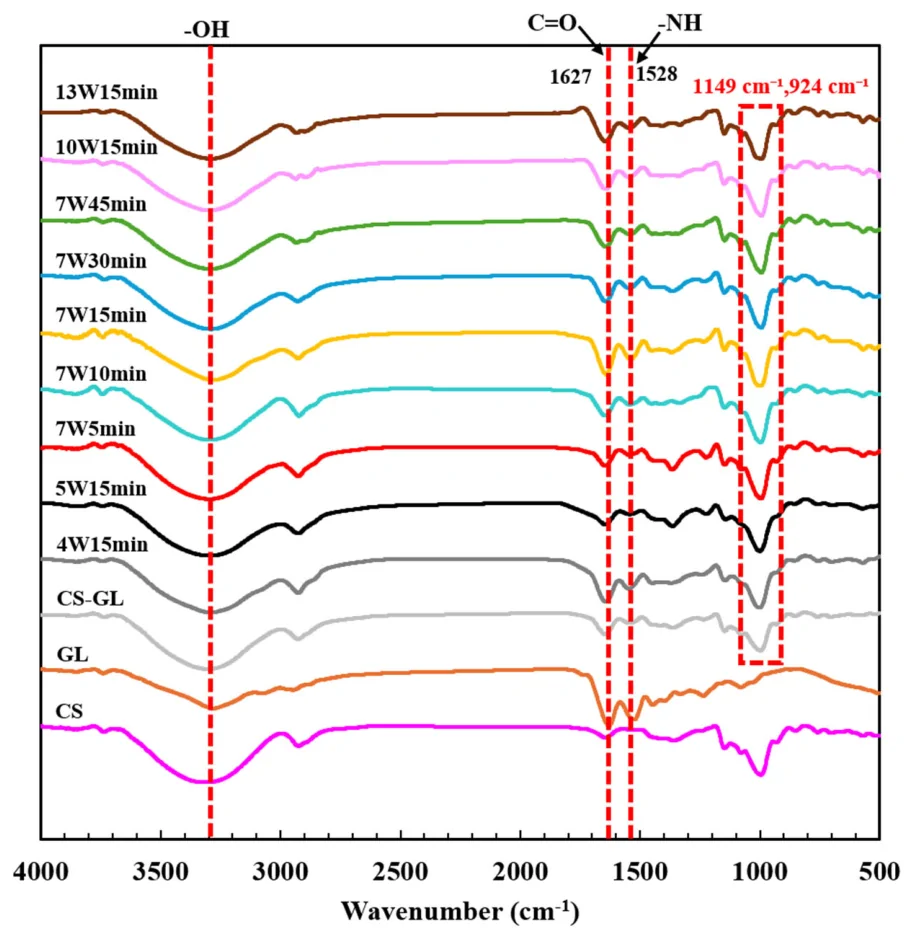
FT-IR of cassava starch film (CS), gelatin film (GL), starch–gelatin blended (CS-GL), and CS-GL plasma-treated films.

The Fourier-transform infrared (FT-IR) spectra of the cassava starch film (CS), gelatin film (GL), starch–gelatin blend film (CS-GL), and plasma-treated CS-GL films are shown in Figure 1. The CS spectrum presented a broad absorption band at 3500–3000 cm^−1^, corresponding to the O–H stretching vibrations of hydroxyl groups, and a C–H stretching band at 2926 cm^−1^ associated with the amylose content [15,16]. The GL spectrum exhibited characteristic protein-related peaks at approximately 1627 cm^−1^ (C=O stretching, amide I) and 1528 cm^−1^ (N–H bending and C–N stretching, amide II) [12]. The CS-GL blend film showed the combined features of both components with slight shifts and changes in band intensity, particularly in the 3500–3000 cm^−1^ and 1630–1520 cm^−1^ regions, arising from hydrogen bonding between the O–H groups of CS and the N–H/C=O groups of GL, which promoted molecular rearrangement and enhanced intermolecular interactions [17].

For the plasma-treated CS-GL films, changes in the band position and intensity were observed relative to those in the untreated CS-GL film. The O–H and N–H stretching bands varied in width and intensity after plasma treatment owing to changes in the hydrogen-bonding environment within the film matrix [12]. The changes observed around the amide I and II regions associated with modifications in carbonyl-containing and amide-related interactions. It has been reported that C=O bands (1700–1600 cm^−1^) are not clearly resolved under argon-gas plasma because of the crosslinking of α-D-glucose [18]; accordingly, the region around 1600 cm^−1^ showed increased and shifted absorption. The intensities of the amide I (1627 cm^−1^, C=O stretching) and amide II (1528 cm^−1^, N–H bending and C–N stretching) bands increased after plasma treatment across the various times and powers with hydrogen bonding between the hydroxyl groups of CS and the carbonyl groups of GL [12,19]. These changes reflected modifications in the local molecular environment of the gelatin chains [12]. The absorption bands at 1200–1000 cm^−1^ correspond to the C–O and C–O–C stretching vibrations of the starch molecules. Changes in the bands at 1149 cm^−1^ and 924 cm^−1^ have been associated with structural modification of starch following plasma treatment [11,20,21]. The changes in these bands suggested modifications in the C–O–C of the starch chains and associated with changes in intermolecular connectivity. At 13 W, the reduction and broadening of the C–O–C bands at 1149 and 924 cm^−1^ were consistent with greater structural heterogeneity and possible chain scission [8,11]. These FT-IR results suggest that plasma treatment induces changes in the molecular interactions and structural organization of the CS-GL films, with the extent of these changes depending on plasma and exposure time. The observed spectra changes were consistent with the coexistence of oxidative modification and changes in the intermolecular connectivity. Such modifications are expected to significantly improve the mechanical and physical properties of plasma-treated films.

When the two-treatment series were compared, the C–O–C bands at 1149 and 924 cm^−1^ served as the clearest indicators of crosslinking. In the power series at a fixed time of 15 min, the intensity of these bands increased from 4 to 7 W and reached a maximum at 7 W, indicating the most extensive formation of new glycosidic linkages between adjacent starch chains. At 10 W, the crosslinking bands remained pronounced; however, at 13 W, their intensity decreased together with the broadening of the –OH region, reflecting a shift from crosslinking toward C–O–C scission and depolymerization. In the exposure-time series at a fixed power of 7 W, the crosslinking bands increased from 5 to 15 min, while the amide I and II bands that had been suppressed at 5 min were restored, indicating that a minimum plasma dose was required to establish stable starch–gelatin interactions. Beyond 15 min, the bands changed only slightly and eventually weakened at 30–45 min as chain scission became competitive. Considering both series, the 7 W–15 min condition produced the strongest crosslinking signature, namely the highest C–O–C intensity, together with retained amide interactions between the hydroxyl groups of starch and the carbonyl and amide groups of gelatin. Therefore, this condition represents the most favorable balance between new bond formation and structural preservation, which is consistent with the mechanical and thermal results discussed below.

### 3.2. Nuclear Magnetic Resonance Spectroscopy (NMR)

**Figure 2:**
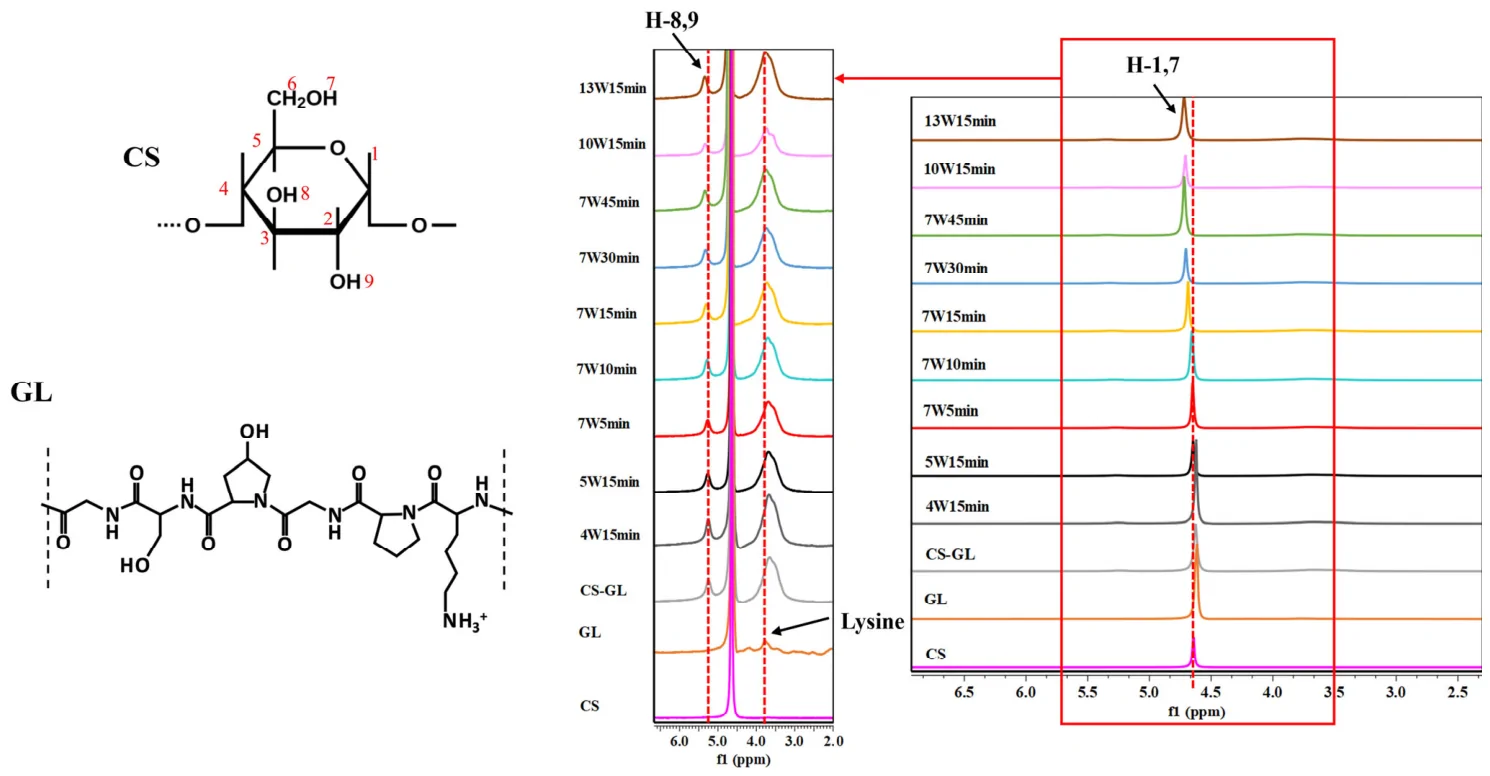
^1^H-NMR spectra of Cassava starch film (CS), Gelatin film (GL), Starch–Gelatin blended film (CS-GL), and CS-GL plasma-treated films.

Figure 2 shows the ^1^H-NMR spectra of CS, GL, the CS-GL blend, and plasma-treated CS-GL films. The CS film exhibited a peak at 4.6 ppm assigned to the ring protons (H-4 and H-5) of the glucose units in the starch backbone. This signal has been reported to be relatively insensitive to crosslinking or depolymerization, indicating preservation of the glucose ring structure [18,20]. The GL film showed signals for the lysine methylene protons (3.7 ppm), the methylene protons (5.3 ppm), and the aromatic amino acid region (7.3 ppm), associated with the –CH groups of the polypeptide backbone, the side-chain protons, and the amide (–NH) protons of the gelatin structure [22,23,24]. Upon blending starch with GL (CS-GL), new peaks appeared at 3.6 and 5.3 ppm, reflecting hydrogen-bonding interactions between the starch and gelatin chains and a more disordered proton environment than in the native films.

**Figure 3:**
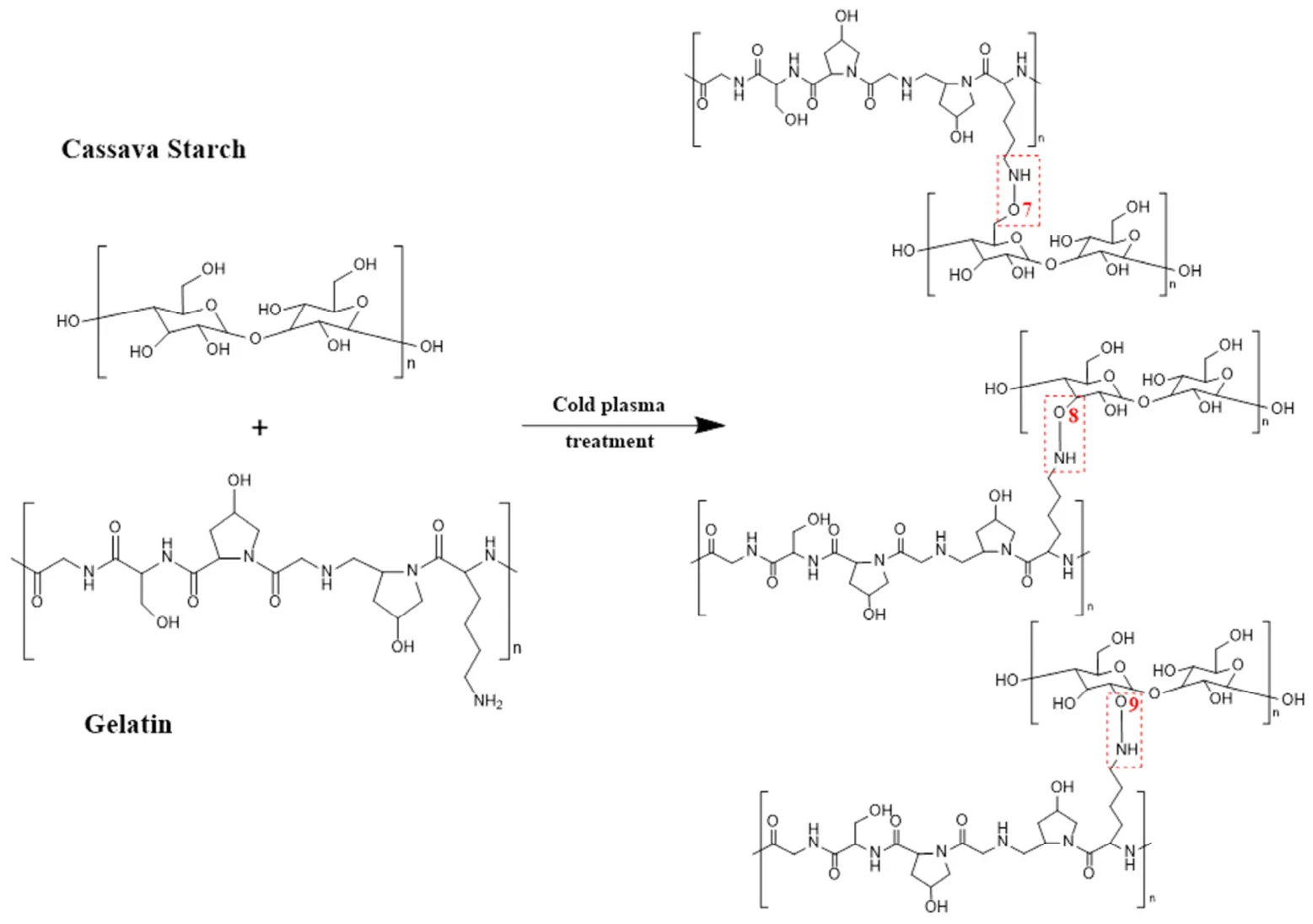
Proposed mechanisms for intermolecular crosslinking after cold plasma treatment of cassava starch and gelatin blending.

After cold plasma treatment, the spectra showed changes in the signal intensity relative to the untreated CS-GL film. At moderate power (4–10 W), the signal intensity in the 3.0–5.5 ppm region decreased, indicating that the functional groups were consumed in crosslinking, which produced a denser and more stable structure. In contrast, high-power irradiation (13 W) yielded broader peaks with reduced intensities, indicating that chain scission and depolymerization were caused by excessive plasma energy. At this power level, the plasma reactive species delivered sufficient energy to cleave C–O–C glycosidic bonds and C–C backbone bonds throughout the bulk of the CS-GL solution, generating polymer fragments of heterogeneous chain lengths. The resulting peak broadening in the 3.0–5.5 ppm region reflects this molecular heterogeneity, as protons in shorter fragments experience different local chemical environments than those in intact polymer chains, producing a broadened rather than a discrete NMR signal. Similarly, increasing the exposure time reduced the intensity, reflecting a decrease in hydroxyl protons [8,18,20]. Thus, the ^1^H-NMR spectra confirm that cold plasma modifies the molecular structure of the CS-GL films through competing crosslinking (Figure 3) and depolymerization mechanisms depending on the plasma power and treatment duration.

A comparison of the two series confirms the same trend. In the power series at 15 min, the reduction in the 3.0–5.5 ppm signals became more pronounced from 4 to 7 W, indicating that an increasing fraction of hydroxyl and backbone protons was consumed in crosslinking; this reduction was greatest at approximately 7–10 W and gave way to peak broadening at 13 W, which is characteristic of chain scission. In the exposure-time series at 7 W, the signal intensity decreased progressively with time as more –OH protons were consumed; however, the peaks began to broaden after 15 min, indicating that prolonged exposure shifted the balance toward depolymerization. Taking both series together, the 7 W–15 min condition again gave the optimal outcome, showing the largest controlled decrease in proton signals without the peak broadening associated with excessive chain scission. This confirmed that crosslinking was maximized, while the polymer backbone remained largely intact under these conditions.

### 3.3. Mechanical Properties

**Figure 4:**
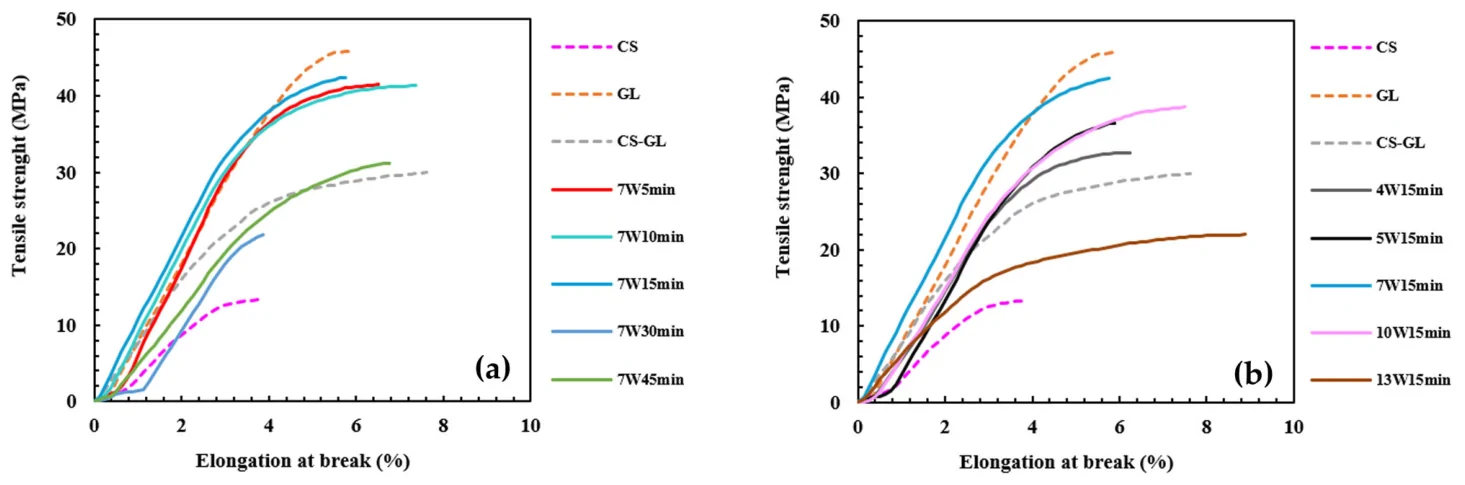
Tensile strength (MPa) and elongation at break (%) of Cassava starch film (CS), Gelatin film (GL), Starch–Gelatin blended film (CS-GL) and CS-GL plasma-treated films. (**a**) Cold plasma treatment at 7 W and (**b**) Cold plasma treatment for 15 min.

The tensile strengths and elongations at break of the CS, GL, CS-GL, and plasma-treated CS-GL films are shown in Figure 4. The CS film exhibited the lowest tensile strength (12 MPa) and elongation at break (4%), whereas the GL film exhibited the highest tensile strength (46 MPa). Blending gelatin into the starch film (CS-GL) increased the tensile strength and elongation at break by approximately 30 MPa and 7%, respectively. This enhancement is attributed to hydrogen bonding and crosslinking between the gelatin and starch molecules, which form a stronger and more cohesive network through interactions between the anionic groups of starch and the cationic groups of gelatin than in pure starch films. Furthermore, starch acts as a crosslinker that reduces molecular mobility by optimizing chain entanglement [17,25].

Cold plasma treatment further improved the mechanical properties of the CS-GL films through crosslinking and etching. At a fixed power of 7 W (Figure 4a), increasing the exposure time from 5 to 15 min increased both the tensile strength and elongation at break. This result was because of hydrogen bonding and plasma-induced crosslinking between the starch and gelatin chains, which improved the structural integrity and resistance to deformation [10,26]. In contrast, longer exposure times and higher plasma powers decreased the tensile strength because of chain damage and molecular weight reduction [15]. Previous studies have reported that plasma treatment renders starch and gelatin films more rigid [27]; consistently, cold plasma treatment in the present study reduced the flexibility of the films while increasing their tensile strength.

The optimal conditions were identified by comparing the two series. In the power series, which was conducted at a fixed time of 15 min (Figure 4b), the tensile strength increased from 4 to 7 W and reached a maximum of 42 MPa at 7 W, after which it decreased at 10 and 13 W. This decrease is explained by the dominance of chain scission at high powers, which reduced the molecular weight faster than crosslinking could reinforce the network. In the exposure time series at a fixed power of 7 W (Figure 4a), the tensile strength increased from 5 to 15 min and then decreased at 30 and 45 min, indicating that prolonged exposure also degraded the chains. The elongation at break followed the opposite trend and decreased as the crosslinking increased, reflecting the reduced chain mobility of the denser network. Considering both series, the 7 W–15 min condition provided the best mechanical performance, delivering the highest tensile strength while maintaining an acceptable elongation. This optimum agrees with the FT-IR and ^1^H-NMR results, which showed the strongest crosslinking signature under the same conditions and confirms that maximum reinforcement was achieved just before chain scission becomes dominant.

### 3.4. Morphology (SEM)

**Figure 5:**
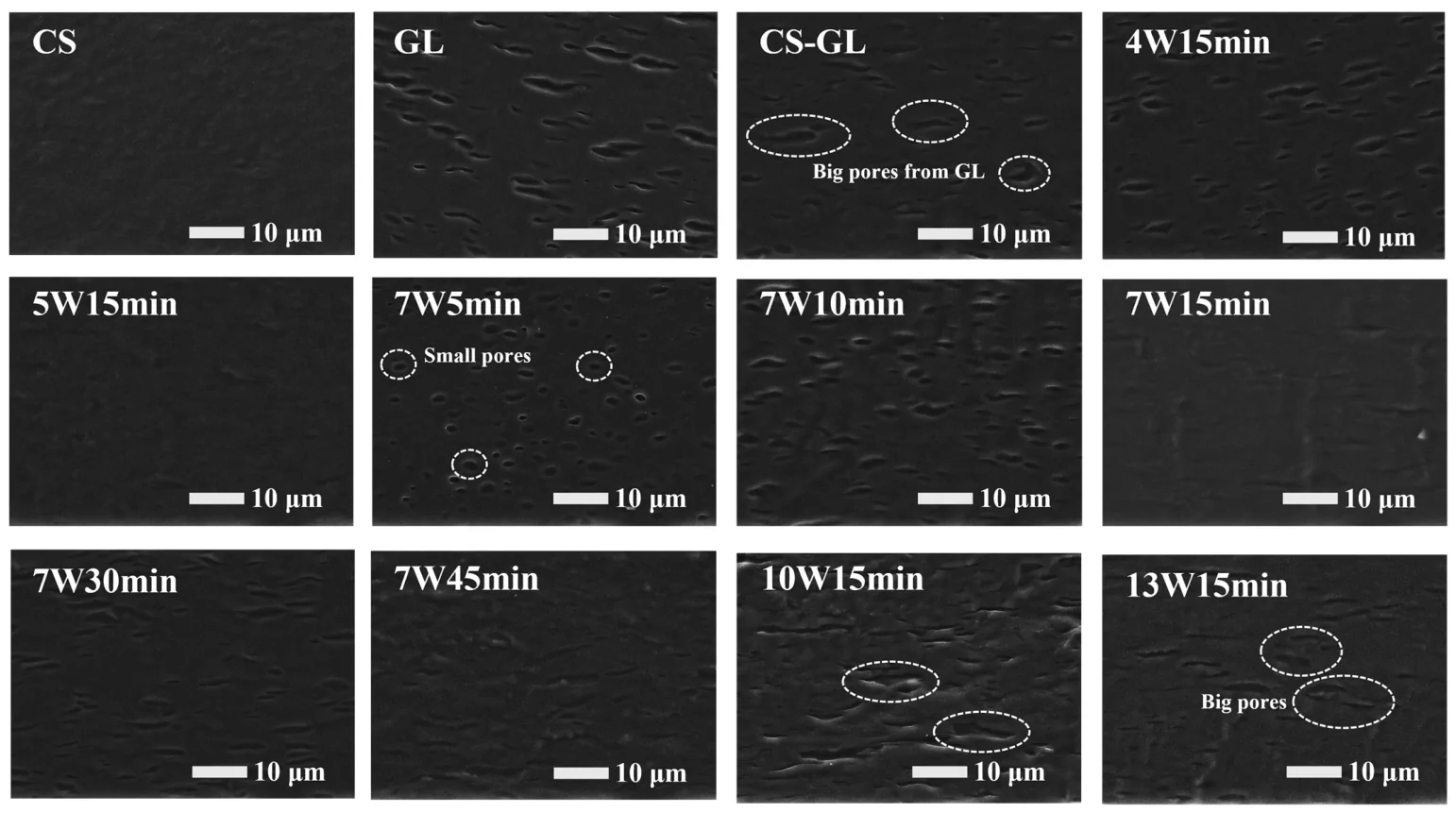
Cross-sectional SEM images of Cassava starch film (CS), Gelatin film (GL), Starch–Gelatin blended film (CS-GL), and CS-GL plasma-treated films. The dotted circles are pores image.

The cross-sectional SEM images of the CS, GL, CS-GL, and plasma-treated CS-GL films are shown in Figure 5. The images were obtained from cryo-fractured specimens and therefore represent the internal cross-sectional morphology. The CS film exhibited a relatively smooth and uniform surface, whereas the GL film showed roughness and pores, indicating that gelatin increased the surface roughness and porosity of the blended films by interacting with the cassava starch chains to form a heterogeneous, porous morphology [17]. After plasma treatment, the films showed distributed small pores and increased smoothness, depending on the plasma power and exposure time, as the cold plasma induced crosslinking of the polymer chains and reduced the amorphous fraction, thereby improving the mechanical properties and hydrophobicity. At a fixed exposure time of 15 min under varying plasma power, the films appeared smoother and denser, indicating enhanced crosslinking between the starch and gelatin chains, which reduced the free volume and limited water penetration. In contrast, at high power (10–13 W), the films exhibited a rougher surface caused by the cleavage of intramolecular bonds, which increased surface hydrophilicity and water absorption. The films treated at 7 W with extended exposure times (30–45 min) showed increased roughness, indicating that prolonged plasma exposure promoted polymer degradation rather than crosslinking [5,20,27,28].

A comparison of the two series supported these property measurements. In the power series at 15 min, the surface became smoother and denser from 4 W to 7 W, whereas at 10 and 13 W, it became rough and porous, consistent with scission-induced degradation. In the exposure-time series at 7 W, the smoothest and densest surfaces were obtained at 15 min, whereas extended exposure (30–45 min) increased the surface roughness. Considering both series, the 7 W–15 min conditions produced the most homogeneous, smooth, and dense morphology, which agreed with its superior mechanical, thermal, and structural properties.

Overall, comparison of all the measured properties across the two-treatment series showed that the 7 W–15 min condition was the best compromise. This condition maximized the tensile strength, the crosslinking signature from FT-IR and ^1^H NMR, the endothermic transition temperature from DSC, and the surface smoothness from SEM while remaining below the power and exposure time at which chain scission became dominant. When water resistance was the primary target, a higher plasma dose was preferable because the lowest swelling and solubility were obtained at 10–13 W or 7 W for 30–45 min and the highest surface hydrophobicity was obtained at 10 W for 15 min. However, these more intensive treatment conditions reduced the tensile strength through chain scission. Therefore, 7 W for 15 min is recommended as the optimal condition for balanced mechanical, thermal, structural, and surface performances, whereas a moderately higher dose can be selected when maximum water resistance is required.

### 3.5. Differential Scanning Calorimetry (DSC)

**Figure 6:**
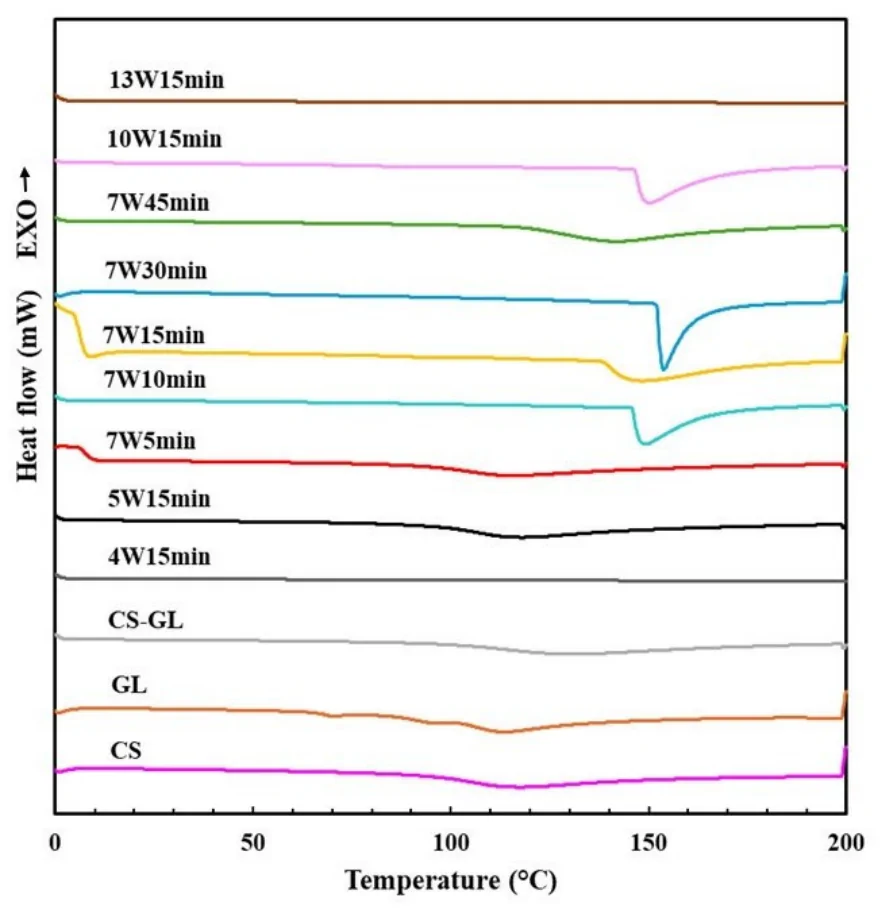
First-heating DSC thermograms of Cassava starch film (CS), Gelatin film (GL), Starch–Gelatin blended film (CS-GL) and CS-GL plasma-treated films.

Figure 6 shows the first-heating DSC scans of the CS, GL, CS-GL, and plasma-treated CS-GL films. The first heating scan reflected the thermal history of the sample and the amount of water bound within the polymer structure, including both free water and water held by hydrogen bonds. The GL film exhibited endothermic peaks at 80–120 °C, indicating that GL, as a protein containing amide, amino, and hydroxyl groups, efficiently forms hydrogen bonds with water; the disordered polypeptide structure results in water loss over a broad temperature range. Similarly, the CS and CS-GL films showed endothermic transitions at 80–120 °C, reflecting the melting of the starch crystalline phase together with the evaporation of water interacting with the –OH and –NH functional groups [17]. Films plasma-treated at moderate power (4–7 W) showed changes in the intensity of these peaks due to crosslinking and structural modification, which was consistent with the FT-IR results showing changes in the intensity of the –OH band, indicating an increase in the melting/endothermic transition temperature. In contrast, increasing the plasma power or exposure time (10–13 W and 30–45 min, respectively) broadened the endothermic peak, indicating depolymerization and an increased proportion of amorphous structure. The narrowing and sharpening of the endothermic transition at moderate plasma conditions were consistent with increased short-range molecular order, while peak broadening under high-power treatment reflects an increase in the amorphous fraction, providing an indirect indication of plasma-induced crystallinity changes. Crystallinity changes were inferred indirectly from DSC endothermic-transition behavior.

Comparing the two series, the endothermic transition temperature increased with plasma power from 4 to 7 W and was highest at 7 W in the 15 min series, indicating a more ordered and thermally stable crosslinked network. At 10–13 W, the transition broadened and shifted, reflecting the increasing amorphous fraction produced by chain scission. In the 7 W series, a similar improvement was observed for up to 15 min, after which the endotherm broadened at 30–45 min. The 7 W/15 min conditions therefore gave the most favorable thermal behavior, consistent with the crosslinking-dominated structure identified by FT-IR and ^1^H NMR spectra. Notably, no clearly resolved endothermic maximum could be identified for the 7 W/45 min and 13 W/15 min samples. This is interpreted as an extreme case of the broadening trend described above: extensive chain scission produces a highly heterogeneous, largely amorphous matrix in which the associated transition is spread over a wide temperature range rather than eliminated, consistent with the depolymerization mechanism proposed throughout this study [16].

### 3.6. Thermogravimetric Analysis (TGA)

**Figure 7:**
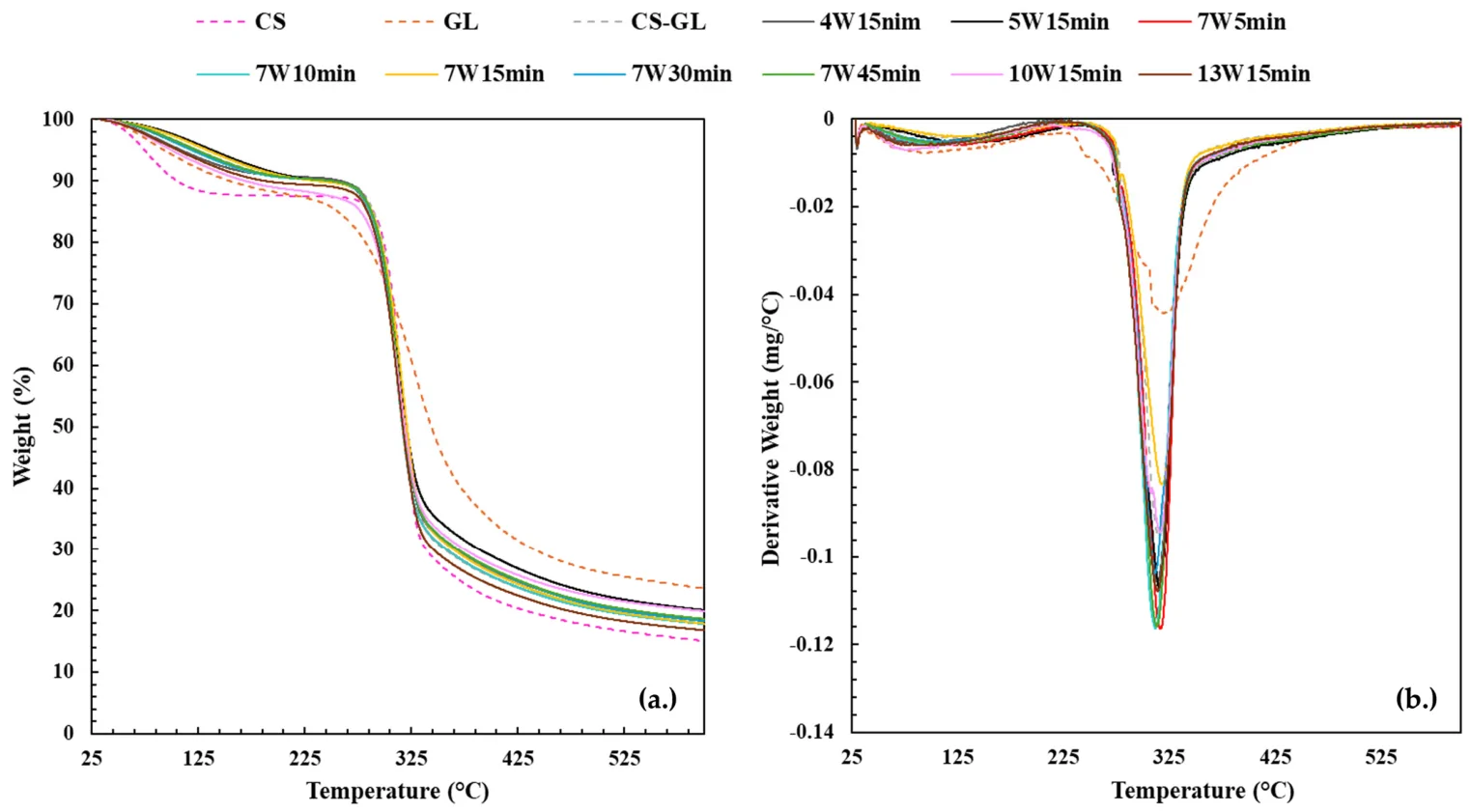
(**a**) Thermogravimetric analysis (TGA) and (**b**) derivative thermogravimetric (DTG) curves of Cassava starch film (CS), gelatin film (GL), Starch–Gelatin blended film (CS-GL) and CS-GL plasma-treated films.

The decomposition temperature and weight loss of the CS-GL and plasma-treated films are shown in the TGA curves (Figure 7a) and derivative (DTG) curves (Figure 7b). The CS-GL and plasma-treated CS-GL films exhibited a single-step thermal decomposition in the range of 300–340 °C. Both the CS-GL and plasma-treated films showed higher residual content than the CS film after decomposition, indicating enhanced thermal resistance resulting from the incorporation of gelatin and plasma-induced structural rearrangement. Plasma treatment alters the decomposition behavior in a manner dependent on both the power and exposure time; within a moderate range, increasing the plasma power (4–10 W) and exposure time (15–45 min) increases the residual content and improves the thermal stability of the films [8].

The rapid weight loss between 250 and 350 °C was attributed to the cleavage of the C–O–C glycosidic bonds of CS and the degradation of the gelatin protein backbone, as cold plasma promotes both chain scission and crosslinking [12,19,20,21]. These behaviors are consistent with the FT-IR results, indicating plasma-induced structural modifications and intermolecular interactions. Above 350 °C, the residual content reflects the stability of the crosslinked network. At moderate power (4–10 W), crosslinking predominated, forming a more thermally stable network, whereas at the highest power (13 W), the residual content fell below that of the untreated CS-GL film, indicating that excessive plasma energy promoted depolymerization and extensive chain scission rather than crosslinking [8,20].

When the two series were compared, the residual content increased with increasing plasma power from 4 to 10 W and then dropped sharply at 13 W, indicating that the char-forming crosslinked network continued to build up until scission became dominant at the highest power. In the 7 W series, the residual content increased with exposure time up to 45 min, consistent with a progressively denser network. It should be noted that the thermal stability continued to improve slightly beyond the point of maximum tensile strength because char formation benefits from a higher crosslink density even when the average chain length is reduced, whereas mechanical strength requires both crosslinking and intact chains. Overall, the highest thermal stability was obtained under moderate to high crosslinking conditions, namely approximately 10 W at 15 min or 7 W with longer exposure, while the 7 W–15 min conditions provided a substantial improvement in thermal stability together with the best mechanical properties.

### 3.7. Water Solubility

**Figure 8:**
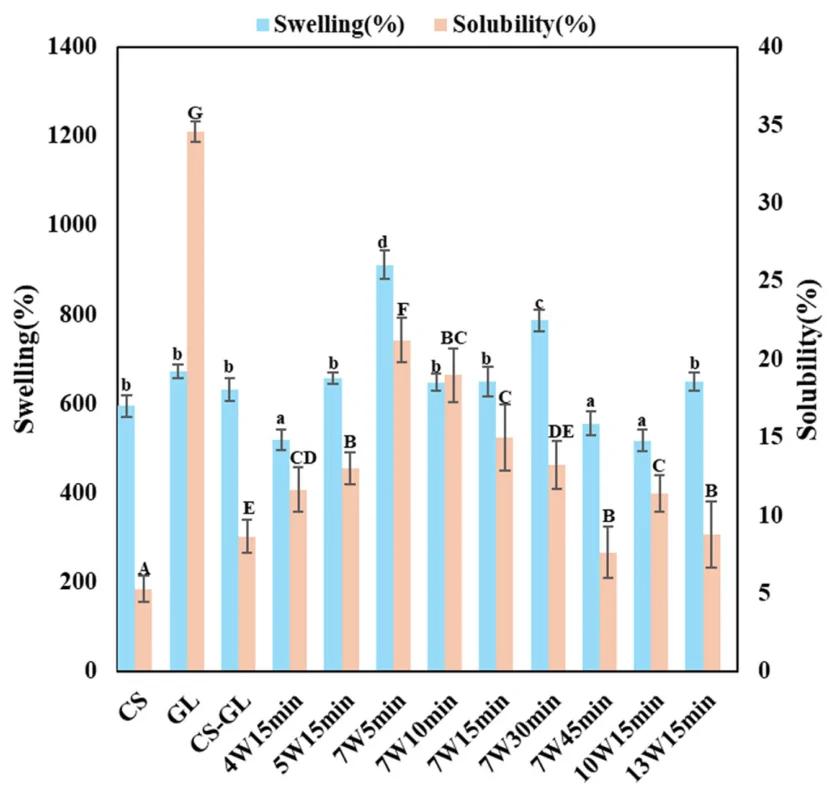
Water swelling (%) and water solubility (%) of Cassava starch film (CS), Gelatin film (GL), Starch–Gelatin blended film (CS-GL) and CS-GL plasma-treated films. Data are expressed as mean ± standard deviation. Different letters denote significant differences between samples (*p* < 0.05), where lowercase letters (a–d) indicate differences in swelling and uppercase letters (A–G) indicate differences in solubility.

The water solubilities of the CS, CS-GL, and plasma-treated films are shown in Figure 8. The lowest solubility (5%) was observed for the CS film. Blending with gelatin (CS-GL) increased the solubility to 9%, indicating that gelatin increases the water solubility of starch-based films by providing additional hydrogen-bonding protons that form new hydrogen bonds between the biopolymer matrix and water molecules [17]. After cold plasma treatment, the solubility varied with the plasma power and exposure time. At a fixed power of 7 W, the solubility decreased with increasing exposure time from 21% at 15 min to 8% at 45 min, which was attributed to crosslinking between the starch and gelatin molecules induced by the plasma-reactive species, reducing the availability of –OH groups that would otherwise interact with water. At a fixed exposure time of 15 min with varying power (4–13 W), the solubility increased with power up to 7 W owing to depolymerization, which broke the starch and gelatin chains into smaller, more water-absorbing fragments; above 7 W, the solubility decreased again as crosslinking between the polymer chains became dominant [27,28,29].

A comparison of the two series clarifies these opposing trends. In the power series at 15 min, the solubility was the highest at 7 W and then decreased at 10 and 13 W; thus, the lowest solubility within this series was obtained at 13 W, where crosslinking dominated. In the exposure-time series at 7 W, the solubility decreased continuously with time and reached its lowest value of 8% at 45 min. Considering both series, the lowest solubility, and therefore the best resistance to dissolution, was achieved under the highest crosslinking conditions, namely 7 W for 45 min or powers above 7 W for 15 min. This optimum differs from that of the mechanical properties because the solubility continues to decrease as the network becomes denser, even when chain scission begins to reduce the tensile strength.

This apparent divergence between the spectroscopic evidence of chain scission at 13 W and the reduced solubility observed under this condition can be reconciled by recognizing that scission and crosslinking are not mutually exclusive but can proceed simultaneously via the same radical-mediated process, with water solubility reflecting the net crosslink density of the cast network rather than the presence of scission at the molecular level. Because plasma was applied to the CS-GL solution rather than a solid film, the radicals and reactive fragments generated by glycosidic bond cleavage retain high mobility and can recombine with reactive sites on adjacent chains before casting, forming new intermolecular crosslinks distinct from the native C–O–C linkages monitored by FT-IR. This interpretation is consistent with the comparatively high-water swelling retained at 13 W (Figure 8) despite the lowest solubility in this series, and with the broadened rather than sharpened DSC transition at this condition, both indicating a structurally heterogeneous but still network-connected matrix rather than a population of freely soluble fragments.

### 3.8. Water Swelling

The water-swelling behavior is shown in Figure 8. The CS-GL and plasma-treated films exhibited significantly greater swelling than the CS film. The CS film showed the lowest swelling, reflecting the interaction between water and the hydrogen-bonded semi-crystalline starch structure [9,16] whereas the GL film exhibited the highest swelling, owing to the extensive hydrogen bonding between water and the hydrophilic amide, hydroxyl, and amino groups along the polypeptide chains, which led to chain disentanglement and matrix expansion [30,31]. Blending GL with CS (CS-GL) increased swelling relative to the native CS film, indicating enhanced water retention arising from the intermolecular interactions between starch and gelatin, which limited polymer dissolution [32]. After plasma treatment, swelling depended on the plasma power and exposure time. At low power (4–7 W) and short exposure times (5–15 min), swelling increased significantly. Previous studies have reported that low-power plasma reportedly increases swelling through hydrogen bond breaking and the formation of oxygen-containing (–OH and C=O) functional groups that enhance water absorption [28,33]. As the power and exposure time increased (10–13 W and 30–45 min, respectively), the swelling decreased, reflecting the dominance of plasma-induced crosslinking, which formed a denser network that limited chain mobility and water penetration, thereby increasing the film stability in water [28,29].

These two series showed consistent trends. In the power series at 15 min, the swelling increased at low power (4–7 W) and then decreased at 10–13 W; thus, the lowest swelling was obtained at 13 W. In the exposure-time series at 7 W, the swelling was the highest at short times (5–15 min) and decreased at 30–45 min, with the lowest value at 45 min. Considering both series, the lowest swelling was achieved under the most extensively crosslinked conditions, namely, 10–13 W at 15 min or 7 W for 30–45 min, where the denser network restricted chain mobility and water penetration. Similar to the solubility results, the best water resistance was obtained at a plasma dose higher than the mechanical optimum, reflecting the tradeoff between network density and chain integrity. The non-linear swelling behavior, together with the continuous decrease in solubility, indicates that CHG affected water uptake and dissolution behavior through different mechanisms. Therefore, the observed increase in contact angle was not interpreted as a direct indication of reduced swelling.

### 3.9. Water Contact Angle

**Figure 9:**
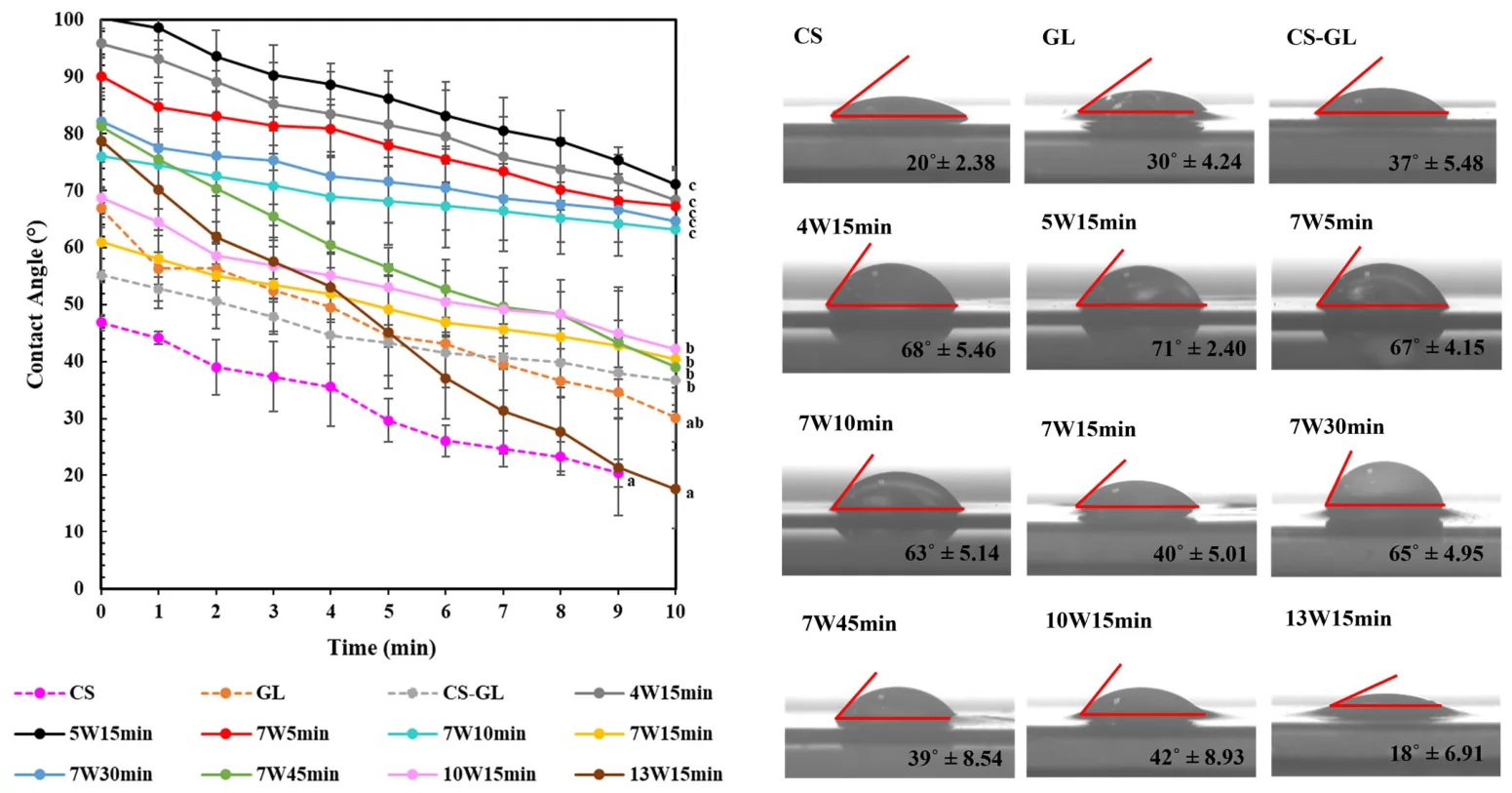
Contact angle (°) of Cassava starch film (CS), Gelatin film (GL), Starch–Gelatin blended film (CS-GL) and CS-GL plasma-treated films. Means with different lowercase letters a–c were significantly different (*p* < 0.05).

The water resistance of the films was evaluated by measuring the water contact angles over 10 min (Figure 9). The contact angles of the CS, GL, CS-GL, and plasma-treated CS-GL films clearly differed, reflecting the differences in surface hydrophilicity. The CS film showed a low initial contact angle that decreased rapidly to 20°, indicating a highly hydrophilic surface due to the presence of hydroxyl and other polar groups on the starch chains. The GL film exhibited a slightly higher initial contact angle than the CS film because of its amide, amino, and hydroxyl groups. Blending GL with CS (CS-GL) produced a higher contact angle than either the CS or GL film alone, indicating enhanced intermolecular interactions and increased hydrophobicity resulting from polymer structure rearrangement [34,35,36].

After plasma treatment, the contact angle increased from 20° (CS and CS-GL films) to 39–71° depending on the plasma power (4–10 W) and exposure time. Previous studies have reported that plasma treatment increases the contact angle and hydrophobicity of starch and gelatin films [27,28]. This increase is consistent with the FT-IR results, which showed a decrease in the –OH band intensity as hydroxyl groups were consumed to form crosslinks between the polymer chains, thereby reducing the surface polarity. However, treatment at 13 W for 15 min produced the lowest contact angle (18°), even lower than that of the untreated films. This finding is attributed to the fact that the high-energy plasma cleaved polymer bonds and generated new oxygen-containing functional groups, particularly –OH; the resulting increase in polar-group density increased the surface energy and lowered the contact angle.

A comparison of the two series reveals the most hydrophobic conditions. In the power series at 15 min, the contact angle increased with power from 4 to 10 W, reached a maximum of approximately 71° at 5 W, and then collapsed to 18° at 13 W, as the high-energy plasma generated new polar –OH groups through extensive bond cleavage. In the exposure-time series at 7 W, the contact angle increased with time as the hydroxyl groups were consumed by crosslinking. In both series, the highest hydrophobicity was obtained at 5 W for 15 min, which was just below the power at which excessive scission regenerated the polar groups. This condition is close to the mechanical and thermal optima, confirming that a moderately high plasma dose maximized the surface hydrophobicity, whereas the highest power was detrimental. The contact angle reflects surface wettability, whereas swelling and solubility represent water uptake and dissolution behavior, respectively. Therefore, these properties exhibited different trends depending on the CHG content.

## 4. Conclusions

This study demonstrates that modifying cassava starch/gelatin (CS-GL) blend films using cold plasma is an efficient and environmentally friendly physical technique, and that the structural, surface, thermal, and water absorption properties of the films can be controlled by adjusting the plasma power and exposure time. FT-IR and ^1^H NMR confirmed that cold plasma induces molecular structural changes. At moderate power (4–10 W), crosslinking predominates through the consumption of hydroxyl (–OH) groups to form new bonds between polymer chains, producing a denser and more stable structure, whereas excessively high power (13 W) leads to chain depolymerization. TGA showed that moderate plasma conditions increased the residual content and thermal stability, whereas high plasma energies reduced the residual content and broadened the thermal transitions, consistent with the DSC results. The plasma dose also governed the surface and water-related properties. Films treated under moderate conditions exhibited a higher water contact angle with reduced swelling and solubility, reflecting increased hydrophobicity due to the reduction of free –OH groups and network densification, whereas films treated at high power showed a rougher and more hydrophilic surface with higher swelling and solubility, as corroborated by SEM. CHG affected the water-related properties of the blends, increasing surface hydrophobicity and reducing water solubility, while the swelling behavior showed a concentration-dependent trend. Overall, cold plasma modification can effectively balance crosslinking and depolymerization in CS-GL films through selective optimization of the treatment conditions, and the resulting films provide a basis for future development of sustainable, biodegradable packaging materials, pending further evaluation of migration safety, biodegradation behavior, and performance under variable humidity.

## Figures and Tables

**Table 1:** The plasma energy (W) and treatment duration (min) for CS-GL plasma treatment.

Materials	Plasma Energy (W)	Treatment Duration (min)	Plasma Dose (J/cm^2^)
4w 15min	4	15	4586
5w 15min	5		5732
7w 15min	7		8025
10w 15min	10		11,465
13w 15min	13		14,904
7w 5min	7	5	2675
7w 10min		10	5350
7w 15min		15	8025
7w 30min		30	16,050
7w 45min		45	24,075

## Data Availability

The original contributions presented in this study are included in the article. Further inquiries can be directed to the corresponding author.
